# Global burden of ischemic heart disease attributable to dietary factors: insights from the global burden of disease study 2021

**DOI:** 10.3389/fnut.2025.1634566

**Published:** 2025-10-02

**Authors:** Yan Wang, Daliang Yan, Wanzi Xu, Bo Min, Zhiwei Fan, Hong Su, Xue Zhao, Dongjin Wang, Yi Zhu

**Affiliations:** ^1^Department of Cardio-Thoracic Surgery, Nanjing Drum Tower Hospital Clinical College of Nanjing University of Chinese Medicine, Nanjing, China; ^2^Department of Cardio-Thoracic Surgery, The Affiliated Huai’an Hospital of Xuzhou Medical University, The Second People’s Hospital of Huai’an, Huai’an, China; ^3^Department of Cardiovascular Surgery, The Affiliated Taizhou People’s Hospital of Nanjing Medical University, Taizhou, China; ^4^Department of Cardio-Thoracic Surgery, Nanjing Drum Tower Hospital, The Affiliated Hospital of Nanjing University Medical School, Nanjing, China; ^5^Department of Pharmacy, The Affiliated Taizhou People’s Hospital of Nanjing Medical University, Taizhou, China

**Keywords:** ischemic heart disease, dietary risk factors, global burden of disease 2021, autoregressive integrated moving average, exponential smoothing model

## Abstract

**Background:**

Dietary risk factors remain a leading modifiable contributor to ischemic heart disease (IHD), yet global trends and inequities in diet-attributable IHD burden remain incompletely quantified. This study examines the global, regional, and demographic burden of IHD attributable to dietary risks from 1990 to 2021, and projects future trends through 2050 using data from the Global Burden of Disease (GBD) 2021 study.

**Methods:**

GBD 2021 estimates were used to quantify IHD-related deaths, disability-adjusted life years (DALYs), years lived with disability (YLDs), and years of life lost (YLLs) attributable to dietary risks across 204 countries and territories. Inequality was assessed using the slope index of inequality (SII) and concentration index (CI). A decomposition analysis evaluated the relative contributions of population growth, aging, and epidemiologic transitions to changes in burden. Future projections were modeled using autoregressive integrated moving average (ARIMA) and exponential smoothing (ES) techniques.

**Results:**

In 2021, IHD attributable to dietary risk factors accounted for 3,906,345 deaths, 89,929,809 DALYs, 1,851,908 YLDs, and 88,077,900 YLLs globally. The highest burden was observed in middle socio-demographic index (SDI) regions. The disease burden was markedly higher in males, with deaths and DALYs peaking at ages 60–64 and 65–69 years. Decomposition analysis revealed that population growth drove a 456.03% increase in global deaths, while accelerated aging in high SDI regions disproportionately contributed to YLDs (−161.51%). Declines in inequality indices suggested reductions in mortality, DALYs, YLDs, and YLLs disparities. Forecasts indicated a continued decline in age-standardized mortality rate (ASMR), age-standardized DALYs rate (ASDR), age-standardized YLDs rate (ASYR), and age-standardized YLLs rate.

**Conclusion:**

Persistent disparities in diet-related IHD burden are shaped by sociodemographic and sex-specific dynamics. Urgent dietary interventions are needed in low- and lower-middle SDI regions, while high-SDI countries must prioritize disability prevention in aging populations. Stratified, context-specific strategies and strengthened monitoring of health inequalities are essential to reduce global cardiovascular disparities.

## Introduction

Ischemic heart disease (IHD), defined as coronary artery disease primarily caused by atherosclerosis, encompasses clinical entities such as stable angina, myocardial infarction (MI), and ischemic cardiomyopathy. According to the Fourth Universal Definition of Myocardial Infarction, MI incidence estimates include both first and recurrent events, while stable angina is characterized as reversible myocardial ischemia induced by exertion or stress, with diagnosis based on clinical presentation. Globally, IHD remains the leading cause of mortality worldwide, accounting for 9.14 million of all deaths in 2019 and contributing to 182 million disability-adjusted life years (DALYs) annually (1, 2). This global burden is shaped by pronounced disparities in socioeconomic development. While age-standardized mortality rates have declined substantially in high-income countries due to decades of preventive strategies and healthcare system investments, low- and middle-income countries (LMICs) continue to experience rising ischemic heart disease (IHD) burdens (3, 4). This growing disparity is compounded by global nutrition transitions, wherein populations, particularly in LMICs, face a dual burden of persistent undernutrition and increased consumption of ultra-processed, nutrient-poor diets (5). The cardiovascular risks associated with these shifts, driven by urbanization and food system globalization, remain insufficiently quantified across different sociodemographic contexts (6, 7).

Although dietary factors are well-established as modifiable drivers of IHD, existing epidemiological models often neglect critical interactions with sociodemographic development, population aging, and gender disparities (8). Regional dietary determinants vary considerably, with high-income countries facing risks from processed and high-sodium foods, while low socio-demographic index (SDI) regions are disproportionately affected by inadequate intake of fruits and vegetables (9, 10). Second, the collinear effects of population aging (global median age increased from 23.6 to 31.0 years since 1990) and dietary shifts remain unquantified, despite evidence that suboptimal diets amplify IHD risks in older adults by 28% (11, 12). Furthermore, biological and sociocultural differences in dietary behavior by sex are rarely incorporated into burden assessments, limiting the precision of public health responses.

This study applies a cross-scale “social-biological-nutritional” framework to examine diet-related IHD burden across 204 countries from 1990 to 2021, using data from the Global Burden of Disease (GBD) study. Through decomposition analysis and sex-specific burden estimation, this study investigates (1) the interaction between dietary risks and sociodemographic development stages, (2) the relative contributions of population aging and dietary exposures to burden trends, and (3) gender-based disparities in DALYs and years lived with disability (YLDs).

## Methods

### Data sources

This study utilized data from the GBD 2021 to assess the disease burden and health inequality trends of IHD attributable to dietary risk factors from 1990 to 2021 (13).^1^ Data covered 204 countries and territories, incorporating mortality, DALYs rates, YLDs rates, and years of life lost (YLLs) rates (14).

The GBD 2021 database integrates multiple sources, including population censuses, civil registration systems, hospital records, epidemiological surveys, and published cohort studies. A standardized approach minimized bias from variations in data collection methods. Mortality estimates were refined using the Cause of Death Ensemble Model (CODEm) to correct for underreporting and misclassification, while non-fatal burden estimates were derived through Bayesian meta-regression modeling (DisMod-MR 2.1) to ensure consistency across regions and periods. Uncertainty intervals (UI) at 95% confidence were calculated using 1,000 Monte Carlo simulations to account for data input errors, model parameter variability, and methodological assumptions (15).

DALYs were computed as the sum of YLLs and YLDs, reflecting total health loss due to disease (16). YLLs were estimated as the difference between actual age at death and the GBD standard life expectancy, while YLDs were calculated based on disease prevalence, disability weights (DW), and disease duration. The SDI classified countries into five categories: high, high-middle, middle, low-middle, and low SDI, based on per capita income, educational attainment, and fertility rates (17).

### Risk factor

In GBD 2021, dietary risks were defined as an aggregate of multiple suboptimal dietary components, including low intake of whole grains, fruits, fiber, legumes, nuts and seeds, seafood omega-3 fatty acids, omega-6 polyunsaturated fatty acids, vegetables, milk, and calcium, as well as excessive consumption of sodium, trans fatty acids, red meat, processed meat, and sugar-sweetened beverages (18).^2^

### Health inequality analysis

To quantify disparities in disease burden across different socio-economic levels, the slope index of inequality (SII) and concentration index (CI) were employed (19). A weighted least squares regression model estimated the association between SDI and disease burden metrics (mortality, DALYs rate, YLDs rate, and YLLs rate). SII represented the absolute difference in burden indicators across the SDI spectrum, while CI measured relative inequality by evaluating the concentration of health indicators in disadvantaged or advantaged populations, constructed based on the Lorenz curve.

### Decomposition analysis

To assess the drivers of changes in the burden of IHD attributable to dietary risks, contributions from population growth, aging, and epidemiological shifts were estimated. The population growth impact was calculated under the assumption of constant age-specific rates, while aging effects were attributed to shifts in demographic structures, such as increasing proportions of elderly individuals. Epidemiological changes were defined as variations in burden driven by alterations in age-specific rates after accounting for the other two factors. Das Gupta’s decomposition method isolated and quantified the contribution of each component (20).

### Future projections

A time series analysis was conducted using the autoregressive integrated moving average (ARIMA) model to forecast the IHD burden from 2022 to 2050 (21). The ARIMA model, denoted as ARIMA (p,d,q), includes three key parameters: p represents the order of the autoregressive (AR) component, capturing the dependency of the current value on past values, d indicates the degree of differencing required to make the series stationary, and q signifies the order of the moving average (MA) component, accounting for the dependency of the current value on past forecast errors. Data stationarity was validated using the augmented Dickey-Fuller (ADF) test, with differencing applied to non-stationary series. The “auto.arima” function in R’s forecast package optimized the model parameters (p, d, q) based on the Akaike Information Criterion (AIC) and Bayesian Information Criterion (BIC). Residual independence was verified through the Ljung-Box test to ensure a white noise distribution. Prediction results were presented with point estimates, and model stability was assessed via rolling-window cross-validation, with root mean square error (RMSE) and mean absolute percentage error (MAPE) computed (22).

The exponential smoothing (ES) model used is the Holt-Winters three-parameter model, which effectively captures long-term trends and seasonal fluctuations (23, 24). The parameters *α* (alpha), *β* (beta), and *γ* (gamma) are key to the model’s functionality. Alpha (α) controls the smoothing of the level component, beta (β) governs the smoothing of the trend component, and gamma (γ) manages the smoothing of the seasonal component. These smoothing coefficients were optimized by minimizing the Root Mean Square Error (RMSE) to enhance the model’s accuracy. The predictive performance of the ES model was compared with that of the ARIMA model, and the final model selection was based on superior Akaike Information Criterion (AIC) and Bayesian Information Criterion (BIC) values, ensuring the chosen model’s robustness and reliability for forecasting.

### Statistical analysis

Estimated Annual Percent Change (EAPC) is a measure used to assess the trend of global IHD attributable to dietary risk indicators, such as age-standardized mortality rate (ASMR), age-standardized DALYs rate (ASDR), age-standardized YLDs rate (ASYR), and age-standardized YLLs rate (25). All analyses were conducted using R (version 4.4.1).

## Results

### Global trends

In 2021, global IHD attributable to dietary risks for deaths, DALYs, YLDs, and YLLs were 3,906,345 (95% UI: −63,184 to 6,213,425), 89,929,809 (95% UI: −2,083,545 to 137,501,226), 1,851,908 (95% UI: 2,518 to 3,267,237), and 88,077,900 (95% UI: −2,087,167 to 134,627,116), respectively (Table 1). Between 1990 and 2021, ASMR, ASDR, and age-standardized YLLs rate declined, with EAPC of −0.88 (95% CI –1.21 to −0.55), −0.91 (−1.14 to −0.68), and −0.93 (−1.16 to −0.70), respectively (Supplementary Figure S1).

**Table 1:** Global burden of IHD attributable to dietary risks from 1990 to 2021.

Metric	1990 Number (95% CI)	1990 ASR (95% CI)	2021 Number (95% CI)	2021 ASR (95% CI)	EAPC (95% CI)
DALYs	63,377,588 (1,023,216 to 93,805,450)	1,608.14 (37.45 to 2,404.74)	89,929,809 (−2,083,545 to 137,501,226)	1,049.02 (−24.85 to 1,604.86)	−0.91 (−1.14 to −0.68)
Deaths	2,628,590 (102,650 to 4,032,758)	75.05 (3.06 to 116.86)	3,906,345 (−63,184 to 6,213,425)	46.76 (−0.77 to 74.74)	−0.88 (−1.21 to −0.55)
YLDs	953,048 (37,389 to 1,650,432)	24.32 (0.95 to 42.24)	1,851,908 (2,518 to 3,267,237)	21.46 (0.02 to 37.78)	0.17 (−0.07 to 0.41)
YLLs	62,424,539 (980,749 to 92,560,598)	1,583.82 (36.23 to 2,373.06)	88,077,900 (−2,087,167 to 134,627,116)	1,027.56 (−24.88 to 1,572.37)	−0.93 (−1.16 to −0.70)

ASR, age-standardized rate.

### National trends

**Figure 1:**
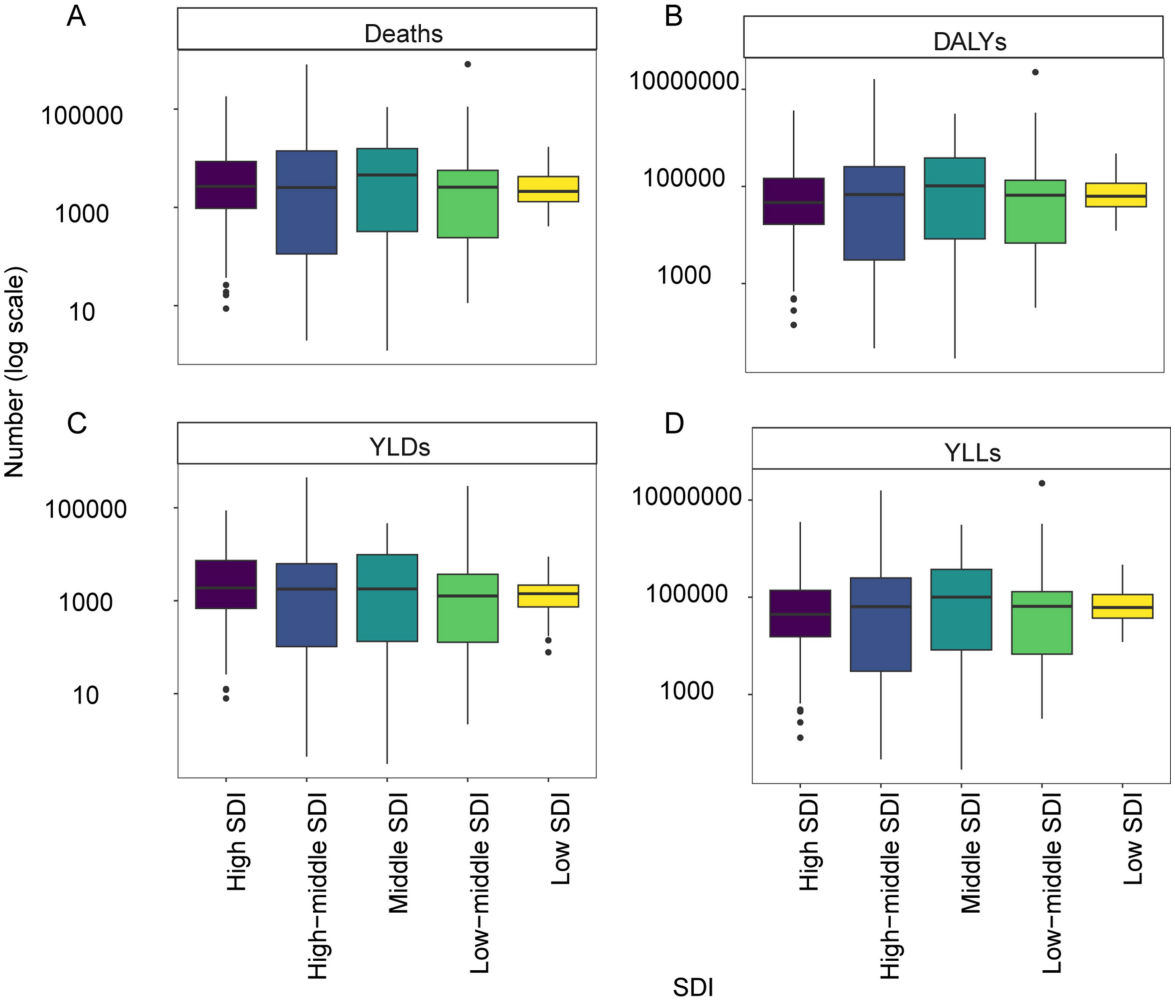
The burden of IHD attributable to dietary risks in 5 SDI regions in 2021. **(A)** Deaths. **(B)** DALYs. **(C)** YLDs. **(D)** YLLs.

Among the 5 SDI regions, the highest deaths (1,209,965; 95% UI: −51,697 to 1,954,245), DALYs (28,817,494; 95% UI: −1,607,093 to 45,071,796), YLDs (580,418; 95% UI: −12,661 to 1,038,122), and YLLs (28,237,076; 95% UI: −1,594,433 to 44,223,762) were observed in the middle SDI regions (Figure 1). The greatest ASMR (64.72; 95% UI: −2.94 to 102.46), ASDR (1560.06; 95% UI: −110.49 to 2405.08), and age-standardized YLLs rate (1536.79; 95% UI: −109.38 to 2367.9) were recorded in the low-middle SDI regions, while ASYR (23.58; 95% UI: 0.37 to 41.81) was highest in high-middle SDI regions (Supplementary Figure S2). Between 1990 and 2021, deaths, DALYs, YLDs, and YLLs increased in middle, low-middle, and low SDI regions, while high SDI regions experienced declines in deaths, DALYs, and YLLs (Supplementary Figure S3).

**Figure 2:**
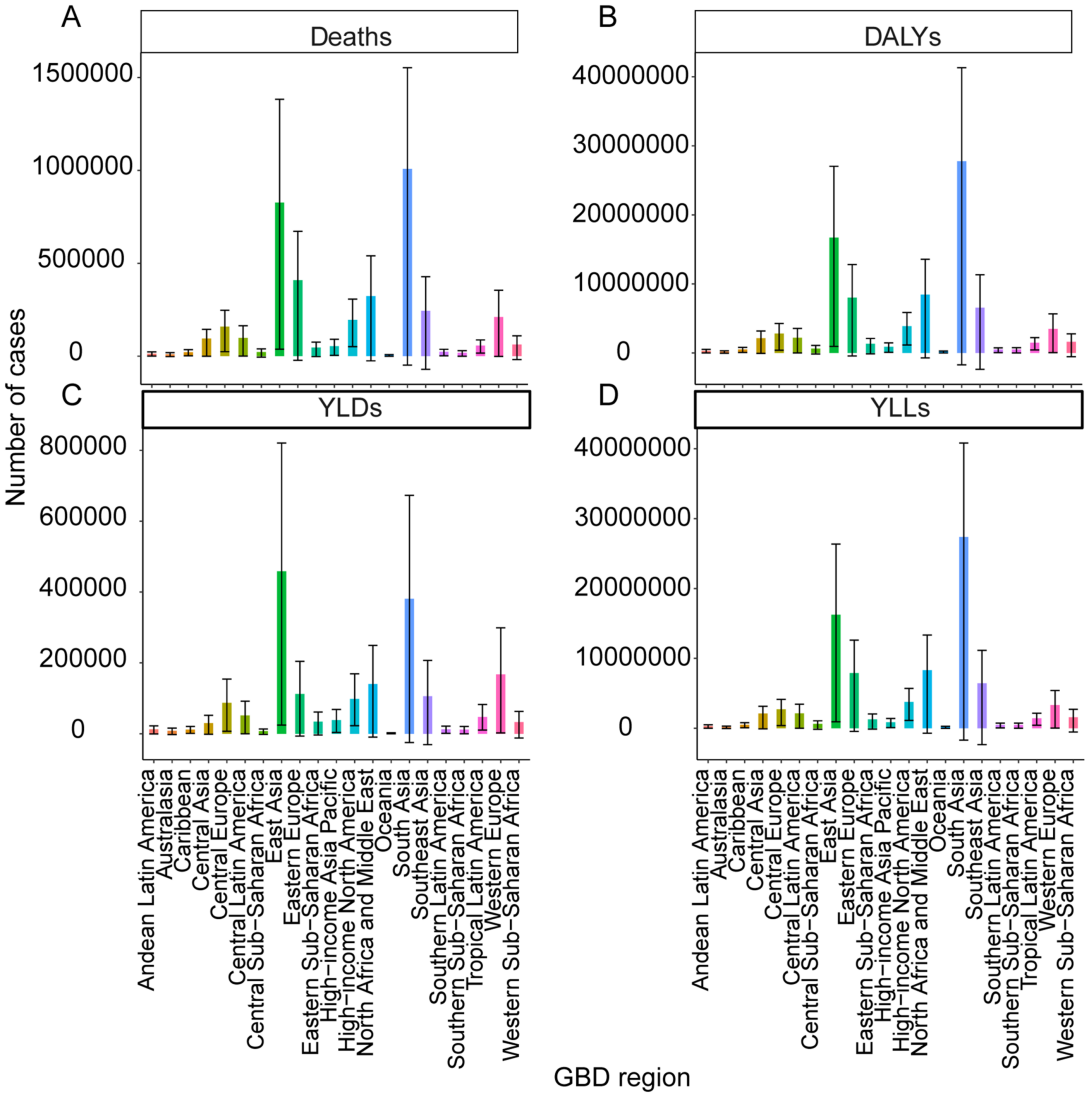
The burden of IHD attributable to dietary risks by SDI regions in 2021. **(A)** Deaths. **(B)** DALYs. **(C)** YLDs. **(D)** YLLs.

At the 54 GBD regions, the highest IHD burden due to dietary risks was in Asia, with 2,359,848 deaths (95% UI: −93,882 to 3,761,329), 57,614,598 DALYs (95% UI: −2,683,199 to 88,611,649), 1,082,416 YLDs (95% UI: −24,064 to 1,932,765), and 56,532,181 YLLs (95% UI: −2,659,135 to 87,073,099). The lowest burden was in Oceania, where deaths, DALYs, and YLDs were recorded at 5,472 (95% UI: −270 to 8,886), 171,137 (95% UI: −19,053 to 278,709), and 1,580 (95% UI: −92 to 2,791), respectively. The lowest YLLs were observed in Australasia (1406.42; 95% UI: −376.88 to 2384.17; Figure 2). Central Asia displayed the highest ASMR of 139.21 (95% UI: 1.22 to 214.03), ASDR of 2,706.01 (95% UI: −49.84 to 4,075.18), and age-standardized YLLs rate of 2,668.32 (95% UI: −48.83 to 4,031.25). Conversely, the lowest values were observed in high-income Asia Pacific, with ASMR at 9.65 (95% UI: 0.93 to 15.84), ASDR at 206.81 (95% UI: 23.14 to 326.92), and age-standardized YLLs rate at 197.37 (95% UI: 22.28 to 312.15; Supplementary Figure S4). Central Europe had the highest ASYR at 39.82 (95% UI: 2.74 to 70.09), while high-income Asia Pacific had the lowest at 9.44 (95% UI: 0.83 to 16.52).

### Countries and territories trends

**Figure 3:**
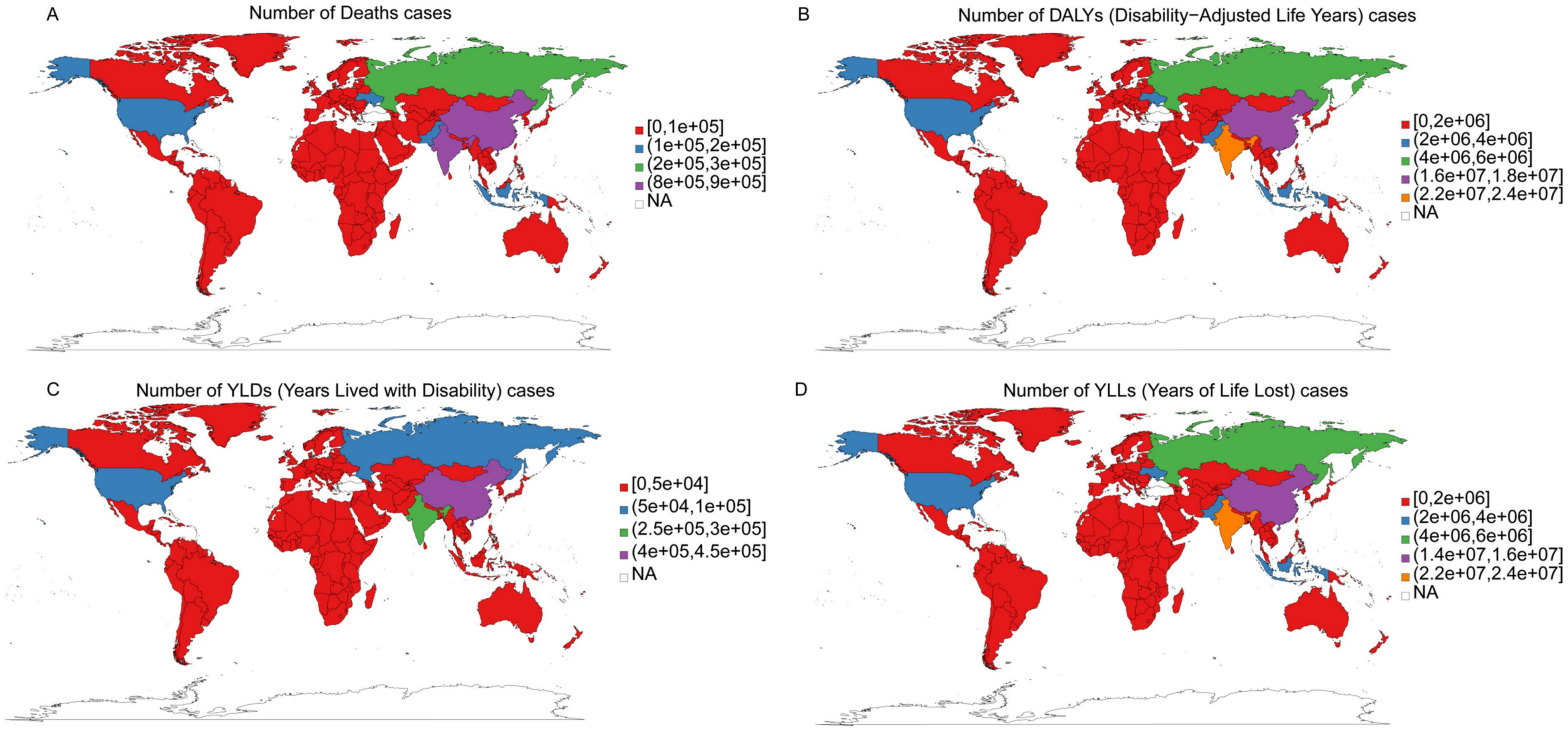
The burden of IHD attributable to dietary risks in 204 countries and territories in 2021. **(A)** Deaths. **(B)** DALYs. **(C)** YLDs. **(D)** YLLs.

India reported the highest numbers of deaths (819,674; 95% UI: −48,479 to 1,273,075), DALYs (22,469,415; 95% UI: −1,645,885 to 33,919,747), and YLLs (22,174,555; 95% UI: −1,623,224 to 33,498,039) attributed to IHD. China had the highest number of YLDs (446,855; 95% UI: 20,516 to 800,408). In contrast, Tokelau had the lowest numbers of deaths (1; 95% UI: 0 to 2), DALYs (29; 95% UI: −4 to 51), YLDs (0; 95% UI: 0 to 1), and YLLs (28; 95% UI: −4 to 50; Figure 3). The highest ASMR was noted in Uzbekistan (181.03; 95% UI: −0.55 to 282.43) and the lowest in San Marino (8.95; 95% UI: 0.27 to 16.7). Nauru exhibited the highest ASDR (4748.75; 95% UI: −996.13 to 8341.3), whereas San Marino had the lowest (182.38; 95% UI: 8.75 to 331.04). Montenegro recorded the highest ASYR (45.11; 95% UI: 3.67 to 80.25), and Brunei Darussalam had the lowest (8.92; 95% UI: −1.31 to 16.82). The highest age-standardized YLLs rate was observed in Nauru (4727.32; 95% UI: −994.17 to 8309.44), while the lowest was in San Marino (165.33; 95% UI: 7.89 to 302.46; Supplementary Figure S5).

From 1990 and 2021, ASMR significantly increased in Lesotho (EAPC: 2.72; 95% UI: 1.69 to 3.76) and declined in Denmark (EAPC: −5.53; 95% UI: −6.34 to −4.71). ASDR exhibited similar trends, rising in Lesotho (EAPC: 3.12; 95% UI: 2.26 to 3.98) and declining in Denmark (EAPC: −5.51; 95% UI: −6.2 to −4.81). The ASYR increased significantly in Guam (EAPC: 2.44; 95% UI: 1.98 to 2.91) and decreased in the United Kingdom (EAPC: −1.92; 95% UI: −2.59 to −1.25). The age-standardized YLLs rate rose in Lesotho (EAPC: 3.17; 95% UI: 2.31 to 4.04) and declined in Israel (EAPC: −5.64; 95% UI: −5.89 to −5.38; Supplementary Figures S6, S7).

### Burden of IHD by age

In 2021, the number of DALYs, deaths, YLDs, and YLLs increased initially with age, then declined. In contrast, ASMR, ASDR, ASYR, and age-standardized YLLs rates rose (Supplementary Figure S8). Conversely, ASMR, ASDR, ASYR, and age-standardized YLLs rates showed an upward trend. Peak DALYs and YLDs were observed in the 65–69 age group, with DALYs at 10,938,146 (95% UI: 62,913 to 17,130,354) and YLDs at 298,164 (95% UI: 12,825 to 556,427). Deaths were most prevalent in the 80–84 age group, totaling 499,962 (95% UI: 4,932 to 851,888).

From 1990 to 2021, an upward trend was observed in deaths, DALYs, YLDs, and YLLs due to dietary risks for IHD increased across all age groups, while age-standardized rates (ASR) decreased. The EAPC for ASMR was −2.24 (95% UI: −2.35 to −2.12), ASDR was −2.25 (95% UI: −2.37 to −2.14), ASYR was −0.89 (95% UI: −0.95 to −0.84), and age-standardized YLLs rate was −2.27 (95% UI: −2.39 to −2.15), with significant declines in the 95–99 age group (Supplementary Figure S9).

### Burden of IHD by sex

**Figure 4:**
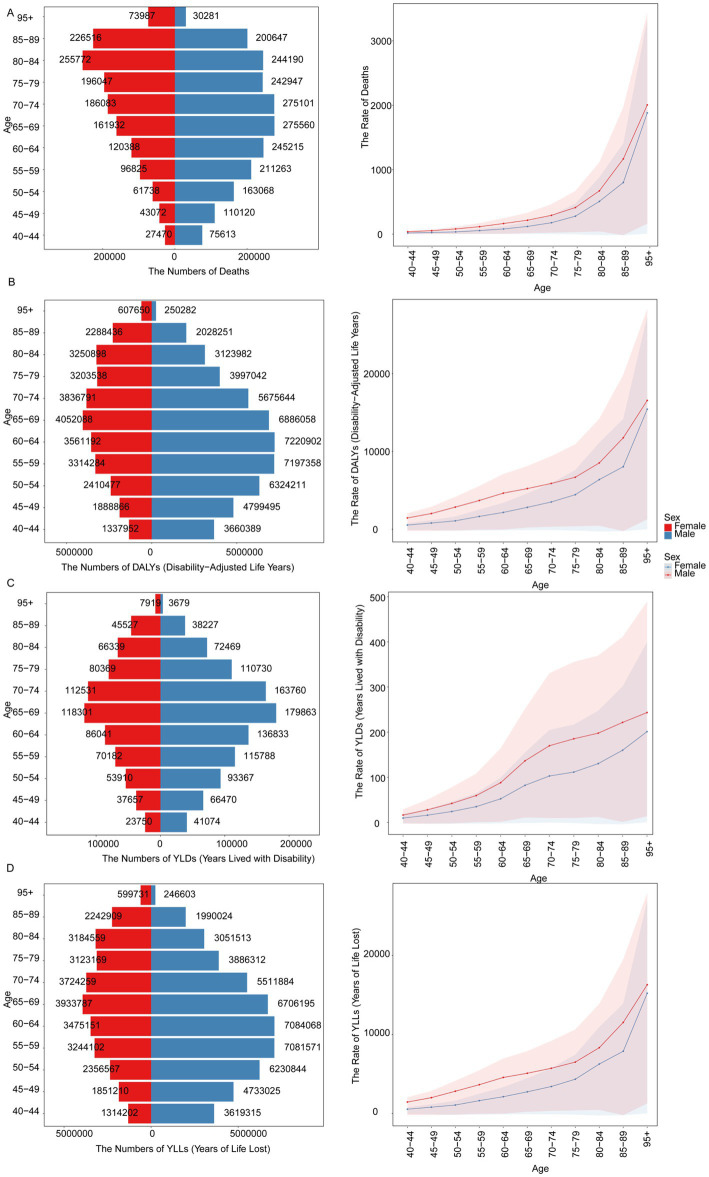
Gender-specific deaths **(A)**, DALYs **(B)**, YLDs **(C)**, and YLLs **(D)** for IHD attributable to dietary risks in 2021.

Regarding the burden of IHD by sex, it was observed that the global disease burden due to dietary risks was higher in males compared to females from 1990 to 2021 (Supplementary Figures S10, S11). In 2021, the number of deaths in males was recorded at 2,263,924 (95% UI: 830 to 3,518,926), whereas in females, it was 1,642,420 (95% UI: −64,806 to 2,753,280). The peak burden of IHD due to dietary risks was observed at different age groups for males and females in 2021. Male deaths were highest in the 65–69 age group, totaling 275,560, while female deaths peaked in the 80–84 age group, at 255,772. Male DALYs were most prevalent in the 60–64 age group, reaching 7,220,902, whereas female DALYs were highest in the 65–69 age group, at 4,052,088 (Figure 4).

### Decomposition analysis of IHD attributable to dietary risks

**Figure 5:**
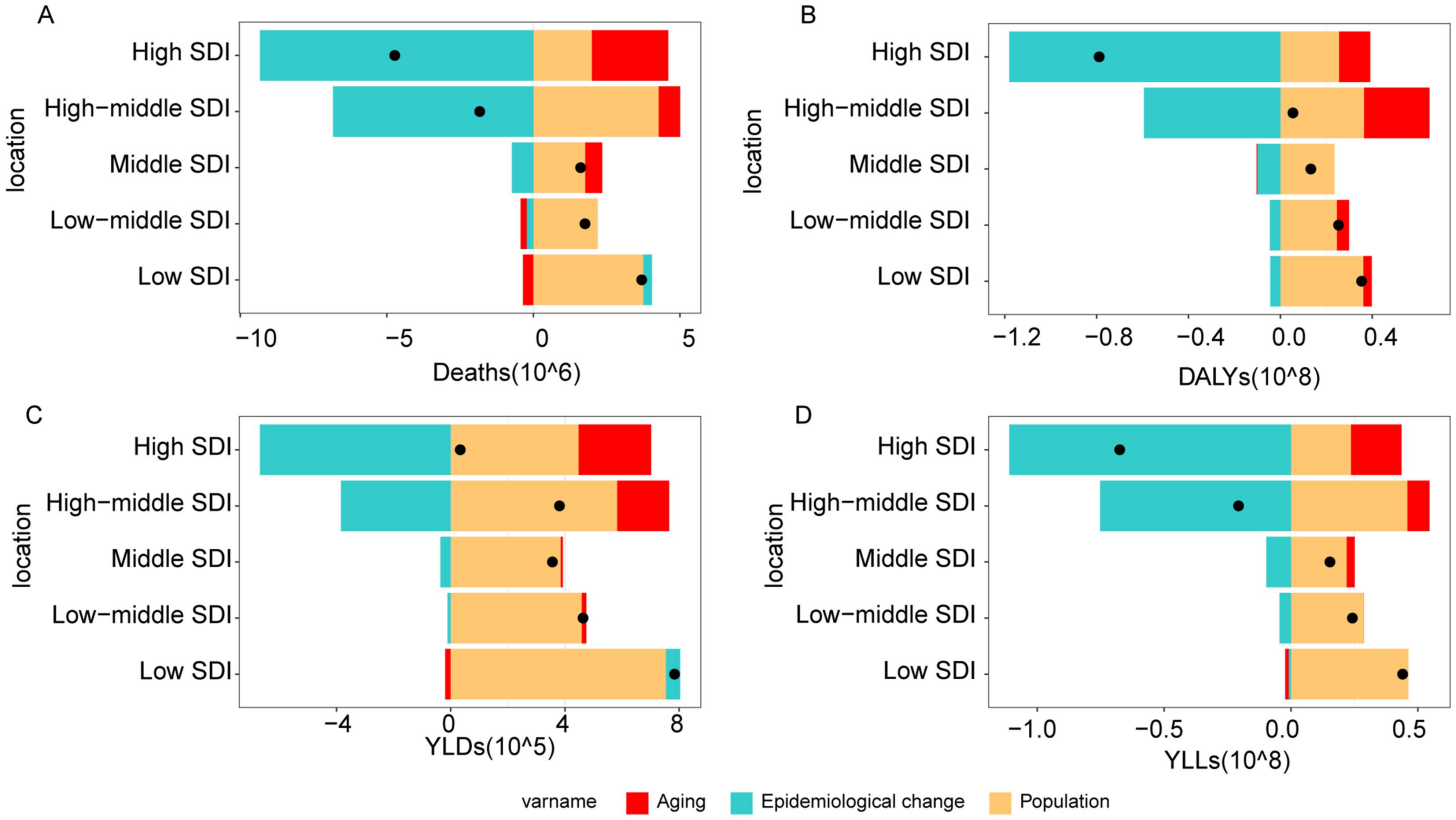
Changes in IHD attributable to dietary risks according to population-level determinants of population growth, aging, and epidemiological change by 5 SDI regions. **(A)** Deaths. **(B)** DALYs. **(C)** YLDs. **(D)** YLLs.

Sex- and SDI-specific analyses of deaths, DALYs, YLDs, and YLLs from IHD attributable to dietary risks demonstrated distinct demographic and epidemiological transition. Global deaths (456.03%) and YLLs (1,306.37%) were predominantly attributable to population growth, while epidemiological transitions accounted for 425.97% of DALYs. Middle SDI regions showed population-driven DALYs (109.74%) but epidemiological transition-mediated YLLs reductions (−53.04%). Low SDI regions exhibited consistent population growth effects on deaths (105.95%), DALYs (104.51%), YLDs (98.79%), and YLLs (107.58%), with epidemiological transitions negatively impacting DALYs (−11.64%). High SDI regions manifested detrimental aging effects, particularly on YLDs (−161.51%) and YLLs (−28.99%; Figure 5).

Population growth disproportionately affected female deaths (911.28%) and YLDs (164.42%), while epidemiological transitions exerted paradoxical effects - reducing female deaths (−1,427.38%) yet amplifying DALYs (329.73%) and YLLs (8,662.08%). Conversely, epidemiological transitions negatively impacted male YLLs (−552.21%), highlighting sex-specific vulnerability patterns (Supplementary Figure S12).

### Health inequality

**Figure 6:**
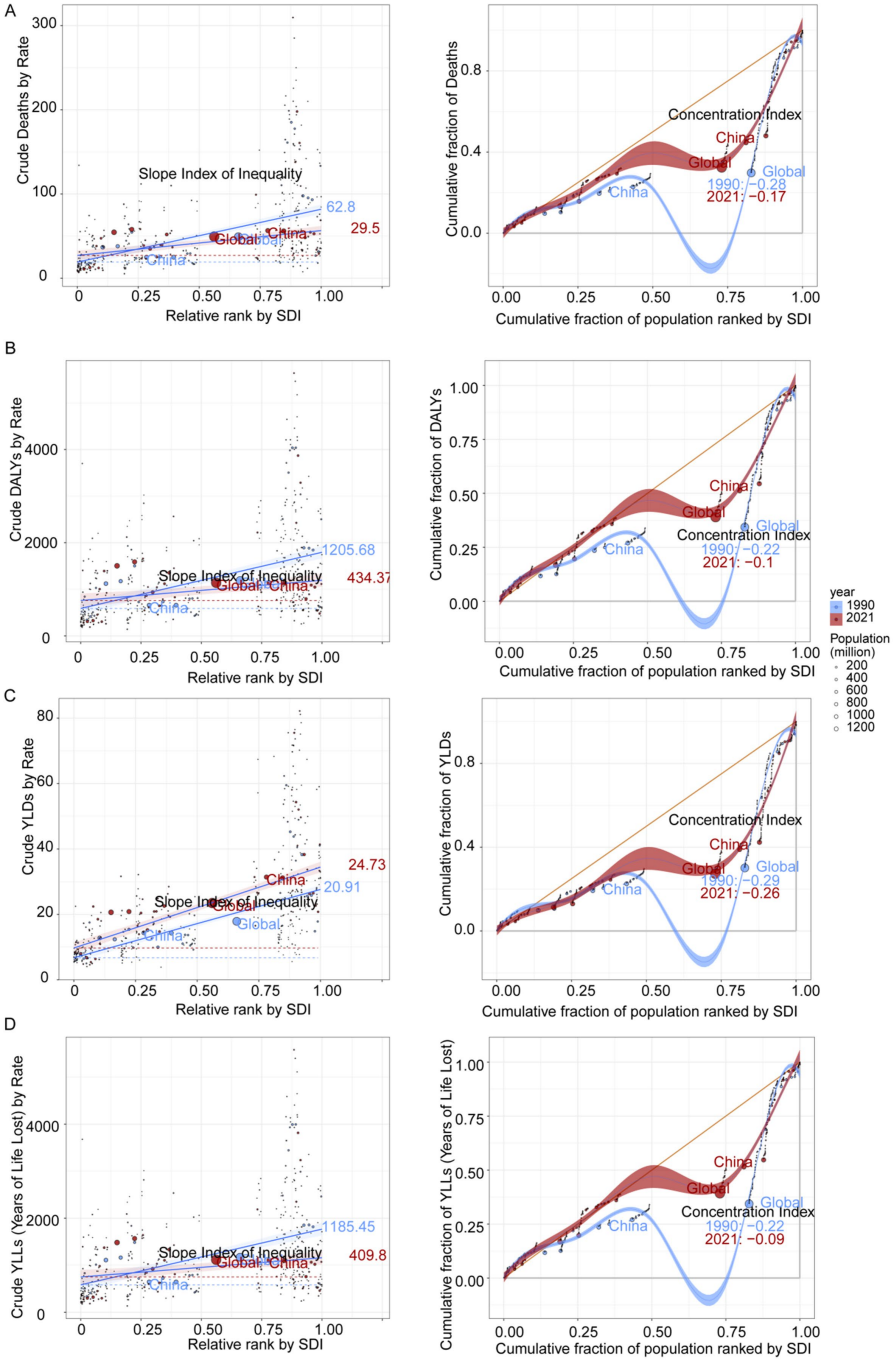
Health inequality regression curves and concentration curves for mortality rates **(A)**, DALYs rates **(B)**, YLDs rate **(C)**, and YLLs rate **(D)** due to IHD attributable to dietary risks in 1990 and 2021.

Between 1990 and 2021, substantial shifts were observed in the distribution of dietary risks associated with IHD. The SII for mortality attributable to IHD decreased from 62.8 to 29.5, while the CI increased from −0.28 to −0.17. These changes indicate a reduction in health inequality on a global scale. Similarly, the SII for DALYs rates dropped from 1205.68 to 434.37, and the CI rose from −0.22 to −0.1. The SII for YLLs rates also declined from 1185.45 to 409.8, with the CI increasing from −0.22 to −0.09. These trends collectively suggest a diminishing degree of inequality (Figure 6).

### Future projections

**Figure 7:**
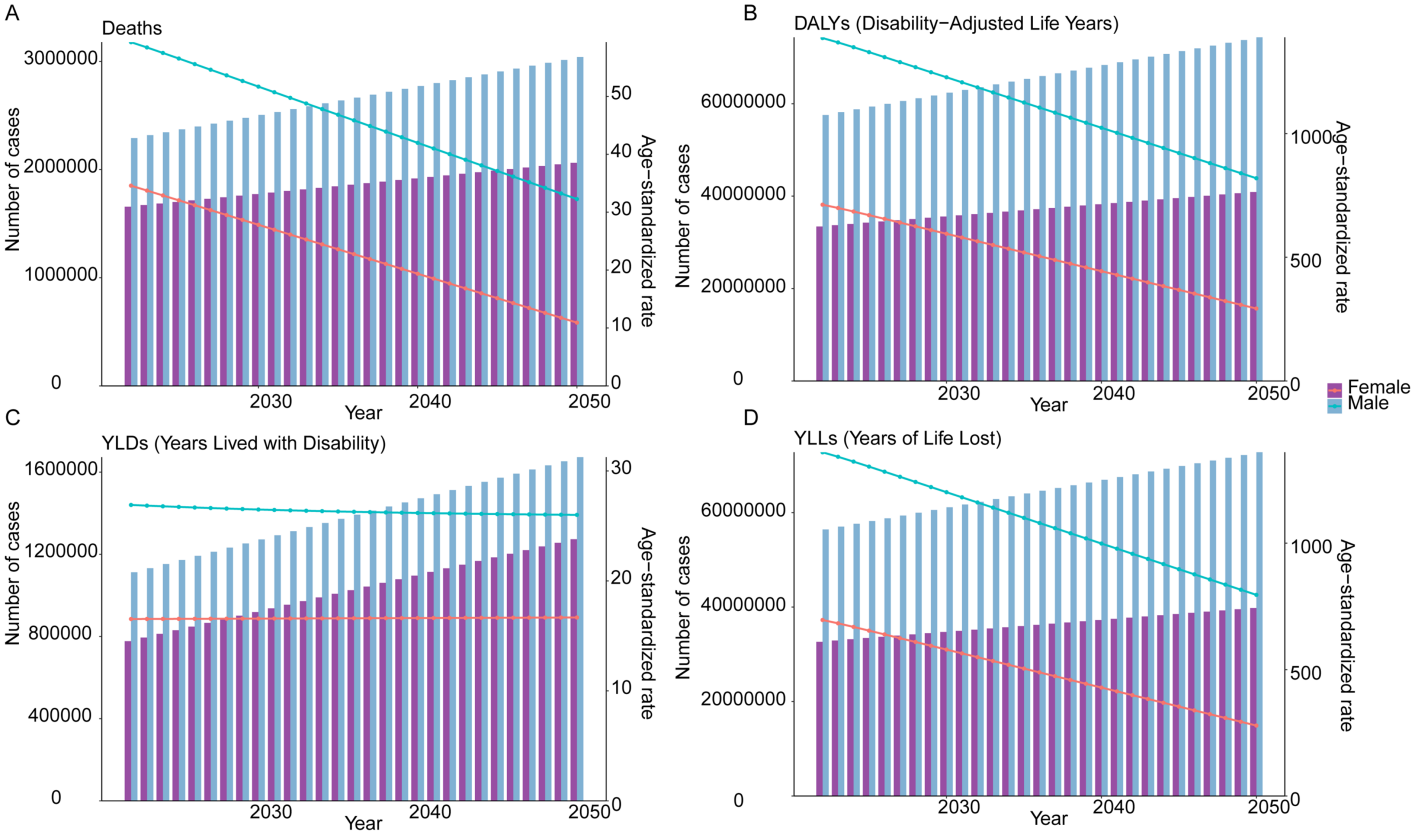
ARIMA model projections for global burden of IHD attributable to dietary risks from 2022 to 2050 by sex. **(A)** Future trends in deaths and ASMR. **(B)** Future trends in DALYs and ASDR. **(C)** Future trends in YLDs and ASYR. **(D)** Future trends in YLLs and age-standardized YLLs rate.

ARIMA modeling projections indicated sustained increases in absolute burden metrics for both sexes between 2022 and 2050. Among males, deaths were projected to reach 3,039,870 (95% UI: 2,866,852 to 3,212,889), with corresponding rises in DALYs (74,370,350; 67,467,288 to 81,273,411), YLDs (1,672,612; 1,433,147 to 1,912,078), and YLLs (72,769,559; 65,855,442 to 79,683,677). For females, deaths were estimated at 2,061,793 (1,926,935 to 2,196,651), accompanied by DALYs (40,882,146; 37,047,786 to 44,716,507), YLDs (1,273,561; 993,830 to 1,553,293), and YLLs (39,783,059; 35,973,209 to 43,592,910). In parallel, ASR demonstrated consistent declines. Males of ASMR were projected to decrease to 32.27 per 100,000 (20.72 to 43.82), with reductions in ASDR (819.68; 486.23 to 1,153.13), ASYR (26.01; 22.21 to 29.81), and age-standardized YLLs rate (795.62; 462.74 to 1,128.50). Female rates exhibited steeper declines in ASMR (10.92; 3.86 to 17.99), ASDR (292.89; 124.08 to 461.69), ASYR (16.68; 9.44 to 23.91), and age-standardized YLLs rate (278.55; 110.66 to 446.44; Figure 7).

**Figure 8:**
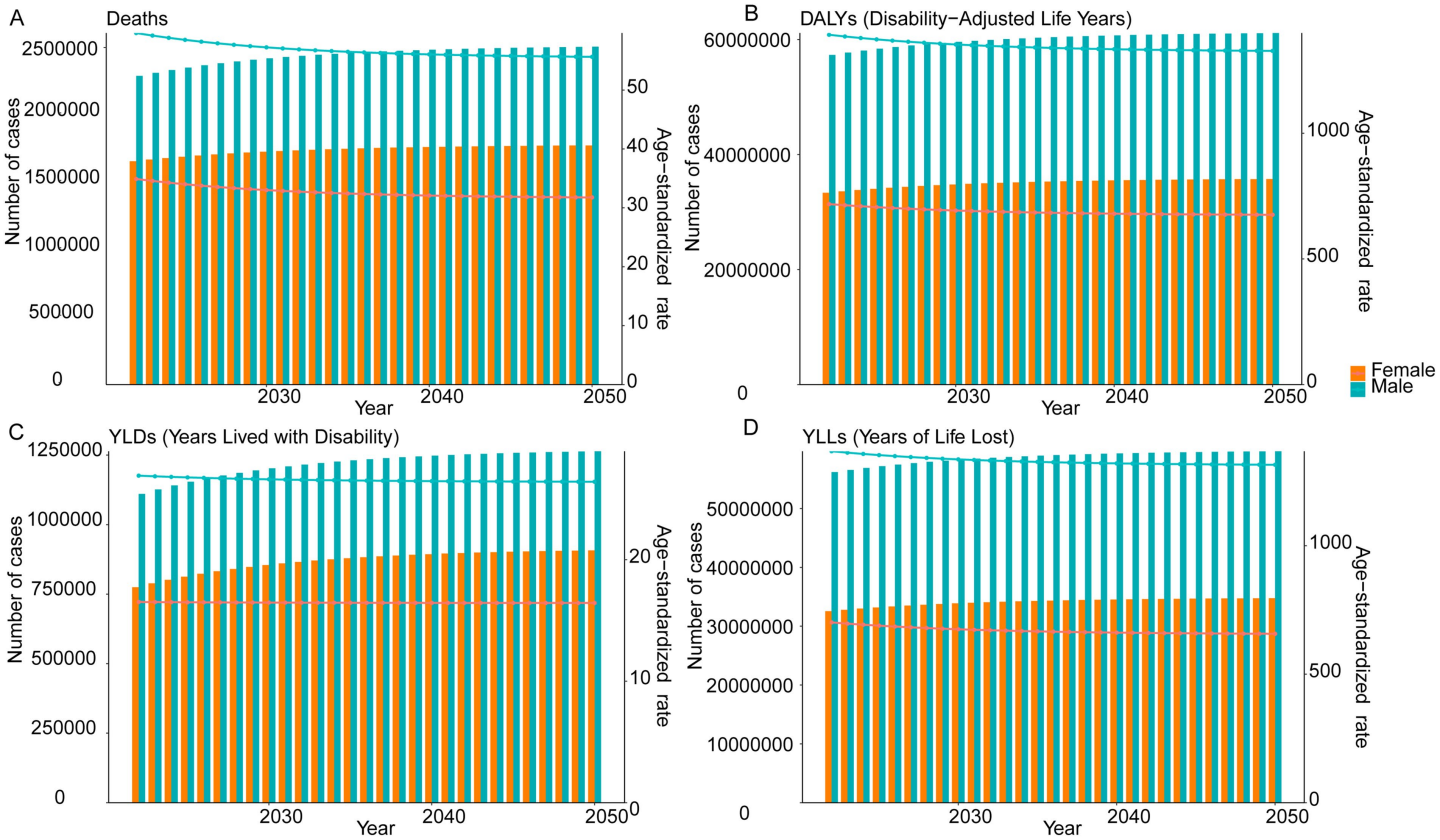
ES model projections of global burden of IHD attributable to dietary risks from 2022 to 2050 by sex. **(A)** Future trends in deaths and ASMR. **(B)** Future trends in DALYs and ASDR. **(C)** Future trends in YLDs and ASYR. **(D)** Future trends in YLLs and age-standardized YLLs rate.

ES models corroborated these trends while highlighting sex-specific divergences. By 2050, male deaths were projected at 2,506,108 (1,632,005 to 3,380,211) with an ASMR of 55.64 per 100,000 (8.61 to 102.66), while female deaths reached 1,773,779 (1,320,211 to 2,227,347) with an ASMR of 31.76 (1.91 to 61.62). Similar patterns emerged for DALYs, with male estimates at 61,118,502 (45,188,508 to 77,048,497) and an ASDR of 1,327.97 per 100,000 (−283.76 to 2,939.71), compared to female DALYs of 35,740,309 (26,367,928 to 45,112,690) and an ASDR of 675.67 (−8.46 to 1,359.80). For male YLDs, projections showed 1,263,983 (1,006,668 to 1,521,298) with an ASYR of 26.42 (23.69 to 29.15), female YLDs of 907,444 (709,594 to 1,105,293) and ASYR of 16.44 (13.90 to 18.98). YLLs estimates further emphasized disparities, with male YLLs reaching 59,747,027 (44,614,026 to 74,880,027) and an age-standardized YLLs rate of 1,314.77 (−432.67 to 3,062.21), compared to female YLLs of 34,763,897 (25,944,346 to 43,583,447) and an age-standardized YLLs rate of 657.11 (18.38 to 1,295.84; Figure 8).

## Discussion

Substantial shifts in the global burden of IHD attributable to dietary risks underscore widening disparities shaped by sociodemographic development, nutritional transitions, population aging, and persistent health system inequities. While ASMR, ASDR, and age-standardized YLLs rates have declined globally, the absolute burden continues to rise, reflecting demographic expansion, dietary westernization, and uneven implementation of preventive interventions. Findings from this analysis reveal regionally distinct trajectories and underscore the need for context-sensitive strategies to mitigate both premature mortality and chronic disability associated with IHD due to dietary risks.

Middle SDI regions had the highest absolute burden, with India alone contributing over one-fifth of global IHD deaths and one-quarter of DALYs (26). This pattern exemplifies the “nutrition paradox,” whereby economic development accelerates the uptake of ultra-processed foods, rising 37% in South Asian markets since 2015, without corresponding gains in the intake of protective dietary elements such as whole grains and fresh produce (27–29). Central Asia exemplifies the adverse convergence of high sodium intake, limited dietary diversity, and underdeveloped prevention programs, with its ASMR exceeding the global average threefold (30, 31). In contrast, high SDI countries such as San Marino and Japan have achieved substantial reductions in IHD mortality, attributable to comprehensive salt-reduction legislation, near-universal statin access, and high adherence to cardioprotective dietary patterns (32).

Stratification by age and sex reveals persistent vulnerability differentials. Males consistently exhibit higher premature mortality, peaking in the 65–69 age group, while females tend to experience higher disability burdens later in life, driven by longer survival and postmenopausal loss of estrogen-mediated cardioprotection (33–35). Sex-specific pathways are further compounded by systemic delays in diagnosis and care. Women in high-income regions experience significantly longer door-to-ECG times, while men in LMICs face elevated occupational exposures and smoking prevalence (36). Decomposition analysis suggests population growth remains the predominant driver of disease burden in low SDI regions, whereas in high SDI regions, aging populations are contributing to disproportionate increases in years lived with disability.

Despite global advancements in acute cardiac care, rehabilitation services remain severely under-resourced. Between 1990 and 2021, inequality in mortality has declined, reflecting the expansion of essential medicine programs across LMICs (37–39). Sub-Saharan Africa, for instance, reports fewer than two cardiac rehabilitation centers per 10 million people (40). Even high SDI regions, while benefiting from low ASDR, face escalating disability burdens due to demographic aging (41).

Forecasts through 2050 indicate that ASMR and ASDR will continue to decline, largely concentrated in middle-SDI countries undergoing rapid urbanization. In China, processed meat intake has increased by 58% since 2005, paralleling a doubling of obesity-related IHD mortality (42). ARIMA and exponential smoothing models project that middle-SDI regions will contribute the majority of future growth in YLLs, driven by demographic momentum and lagging policy responses. Consistent with findings by Shi et al., demographic momentum and epidemiological transition are anticipated to drive the largest future increases in YLLs, reinforcing the vulnerability of countries in nutritional transition. Strengthened food environment regulation, expansion of primary prevention, and population-based dietary interventions represent urgent priorities to mitigate the escalating IHD burden in these settings (6).

To address these trends, three strategic priorities emerge. First, national regulatory frameworks targeting dietary risk factors must be scaled. South Africa’s sodium legislation, which caps salt content in bread at 400 mg per 100 g, has achieved measurable reductions in blood pressure and could reduce IHD mortality by over 12 percent if adopted in similar contexts (43). Second, equity in rehabilitation access must be prioritized through integration with mobile health platforms and social protection systems. Third, high SDI countries must integrate geriatric care into cardiovascular disease management, combining Mediterranean diet protocols, physical activity interventions, and mental health screening to reduce disability among aging populations (44).

Several limitations must be acknowledged. First, reliance on GBD data introduces uncertainties associated with incomplete cause-of-death registration, particularly in low-income countries, which may result in underestimation of the actual burden of IHD. Second, the absence of urban–rural stratification limits the ability to examine intra-national disparities, constraining the precision of geographically targeted interventions. Third, broad regional groupings within the GBD framework may obscure important cultural, social, and health system differences that influence dietary exposures and cardiovascular outcomes (45). Finally, our age-stratified analysis began at 40 years of age. Although GBD data are available for younger populations, the burden of dietary risk for attributable IHD below 40 years is minimal and characterized by wide uncertainty intervals, consistent with previous global and national analyses (10, 46). While younger age groups were excluded, this omission is unlikely to affect our findings, as the vast majority of dietary risk for IHD occurs among adults aged 40 years and older. These constraints underscore the need for localized epidemiological studies to inform context-specific policy responses. Nonetheless, the strong concordance observed across dual-model validation enhances confidence in the robustness and methodological reliability of the findings.

The burden of IHD due to diet demonstrates marked disparities across sociodemographic and gender groups, highlighting the limitations of uniform dietary recommendations and the need for strategies that account for both population-level characteristics and individual-specific factors. Integration of planetary health diets with precision nutrition approaches offers a promising strategy to align dietary interventions with health and sustainability objectives. Evidence indicates that precision nutrition, tailoring dietary guidance based on genetic makeup, gut microbiome composition, metabolic profile, and lifestyle, can enhance cardiovascular disease prevention outcomes (47, 48). Personalized interventions have been shown to improve adherence and cardiometabolic profiles while optimizing resource allocation by focusing on high-risk populations (49). Combining individualized dietary patterns with sustainably sourced plant-based foods may simultaneously address the dual challenges of reducing global IHD burden and promoting environmental sustainability, consistent with the principles of the planetary health diet. Future research should incorporate mixed-methods approaches to explore how food environments, policy contexts, and cultural norms interact to produce cardiovascular risk across diverse settings. The integration of planetary health diets with precision nutrition strategies holds promise for aligning dietary interventions with both health and sustainability goals. Collectively, these findings suggest that the integration of planetary health diets with precision nutrition strategies provides a feasible and impactful pathway to optimize population and individual health interventions while advancing sustainable dietary practices and mitigating IHD burden.

## Conclusion

In summary, the burden of IHD due to dietary risks reflects both progress and persistent gaps in global cardiovascular health equity. Effective response will require stratified approaches: scaling regulatory reforms and preventive care in middle SDI regions, while prioritizing disability mitigation and healthy aging in high-income regions. In addition, low SDI regions require urgent attention, as the persistent high burden and continued population growth amplify the effects of dietary risks. Strengthening primary prevention through affordable nutrition policies, improved food security, and equitable access to essential cardiovascular care will be critical in these settings. Multisectoral collaboration is essential to bridge the divide between clinical care, public health, and sustainable food systems.

## Data Availability

The datasets presented in this study can be found in online repositories. The names of the repository/repositories and accession number(s) can be found at: https://ghdx.healthdata.org/gbd-2021.

## Footnotes

^1^ https://ghdx.healthdata.org/gbd-2021

^2^ https://www.healthdata.org/research
