# Supplementary material for: Global burden of ischemic heart disease attributable to dietary factors: insights from the global burden of disease study 2021
Source: Front Nutr. 2025 Oct 2;12:1634566. doi: 10.3389/fnut.2025.1634566 (PMC12527891; doi:10.3389/fnut.2025.1634566)
Supplement: Supplementary file 1 [file Image_1.pdf]

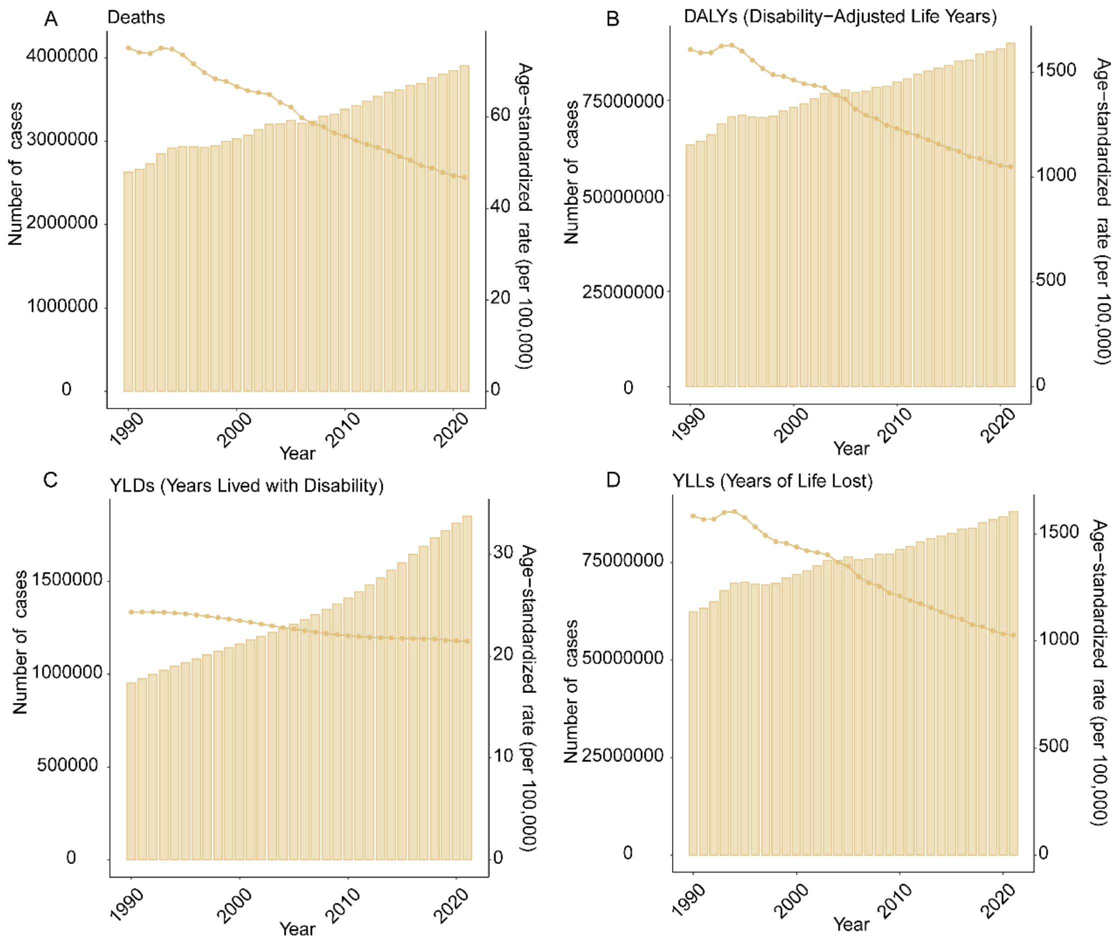


Figure S1 Global burden of IHD attributable to dietary risks from 1990 to 2021. (A) Deaths and ASMR. (B) DALYs cases and ASDR. (C) YLDs cases and age-standardized YLDs rate. (D) YLLs cases and age-standardized YLLs rate.

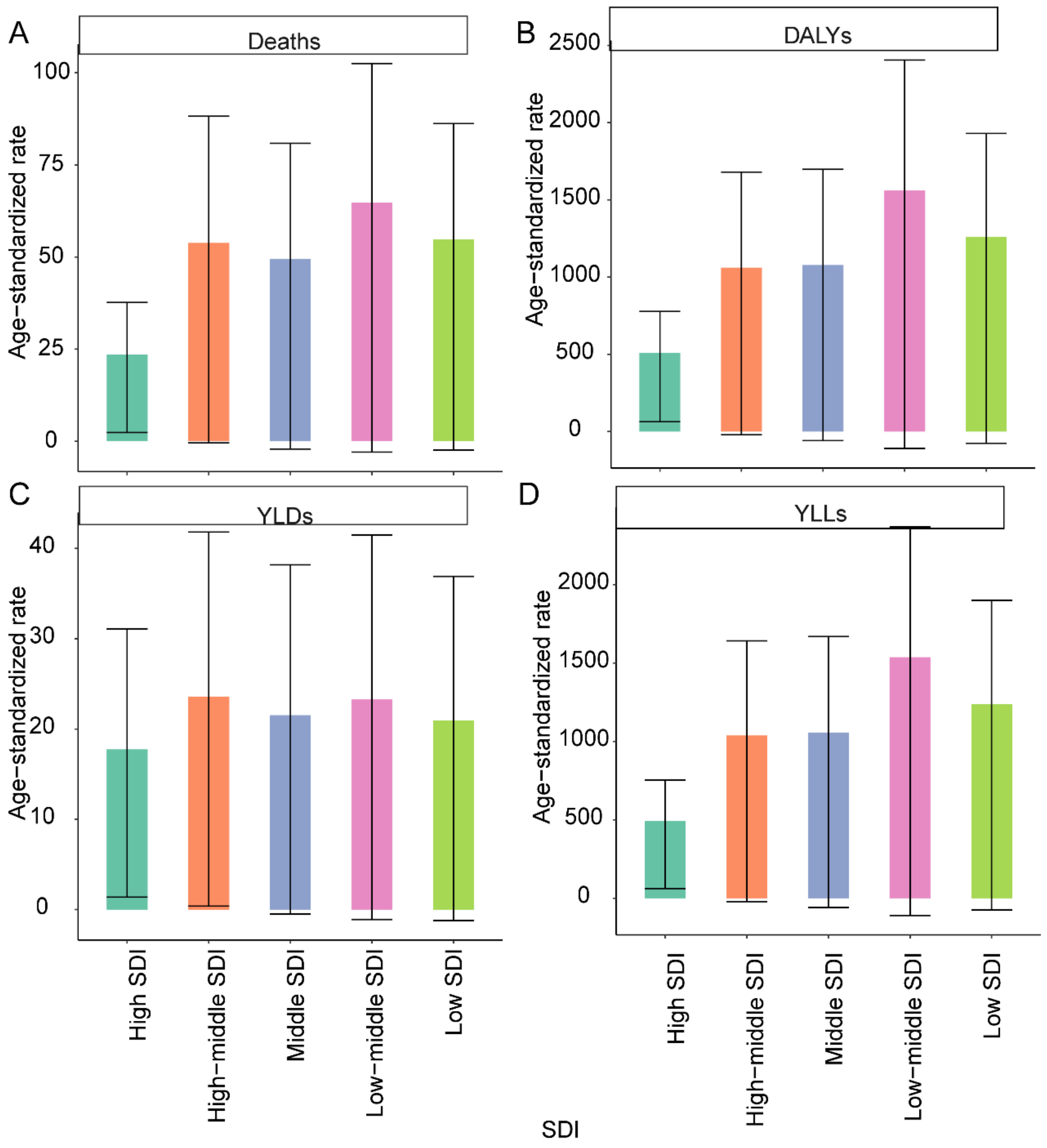


Figure S2 The burden of IHD attributable to dietary risks in 5 SDI regions in 2021. (A) ASDR. (B) ASMR. (C) Age-standardized YLDs rate. (D) Age-standardized YLLs rate.

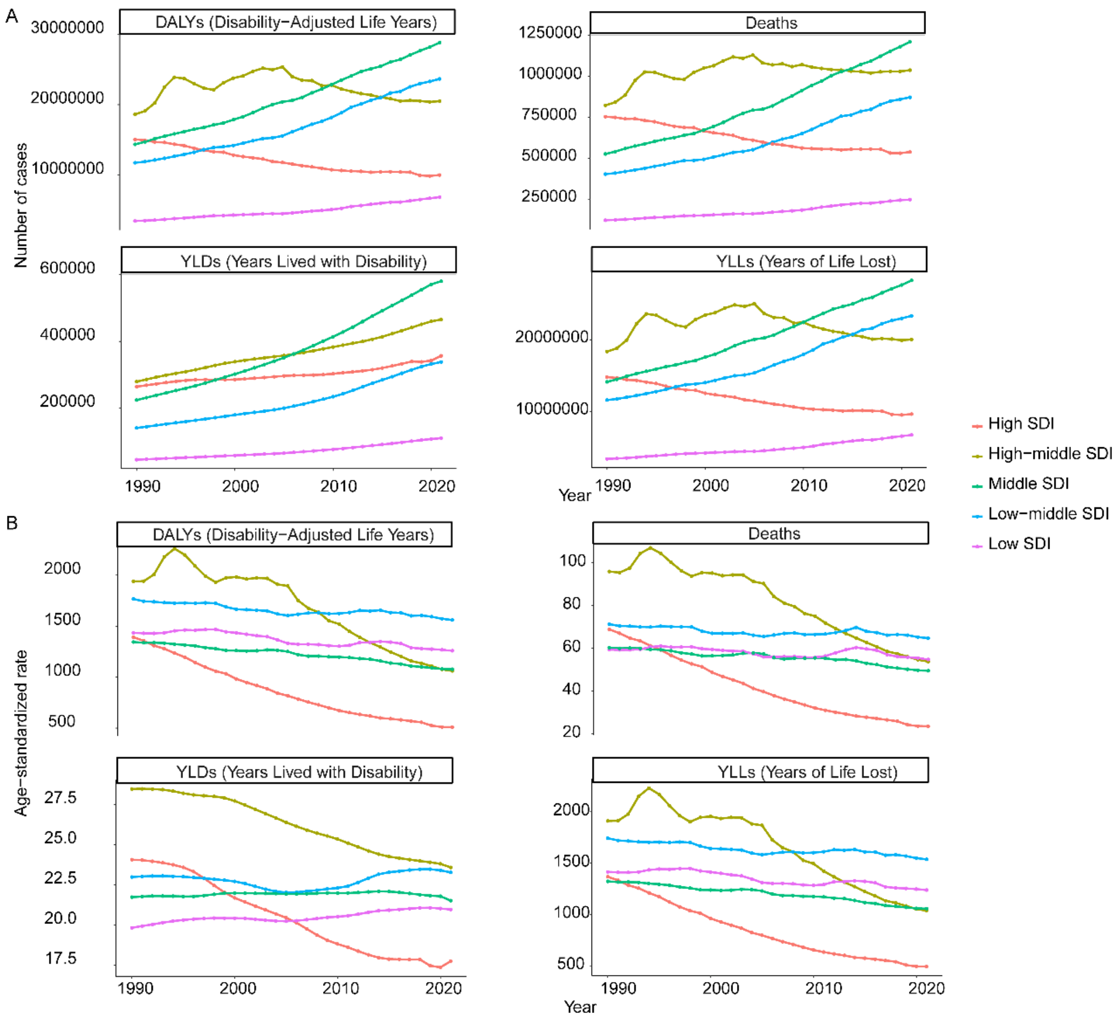


Figure S3 Trends in IHD attributable to dietary risks by SDI regions from 1990 to 2021. (A) The number of deaths, DALYs, YLDs, and YLLs. (B) ASMR, ASDR, age-standardized YLDs rate, age-standardized YLLs rate.

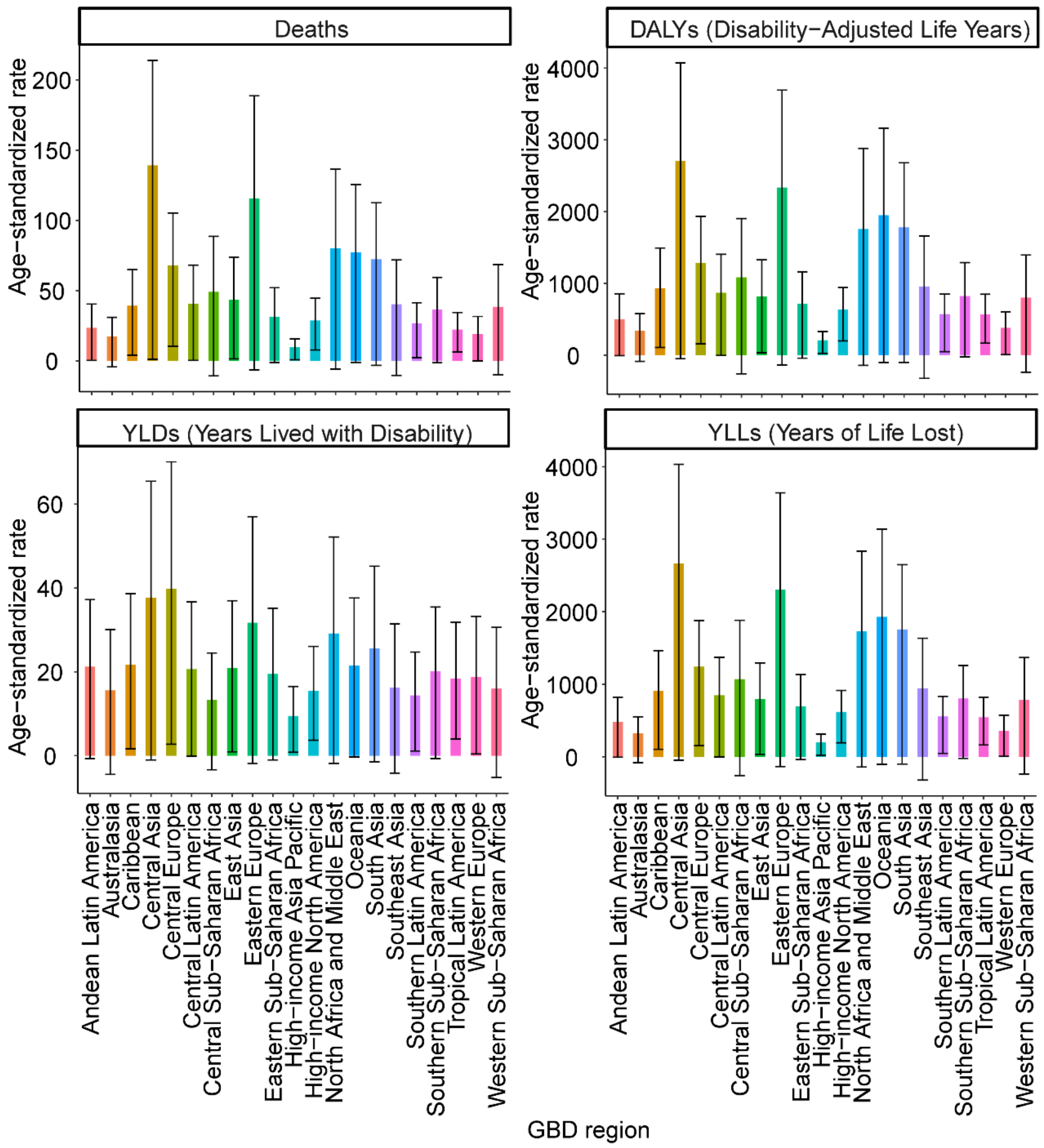


Figure S4 The burden of IHD attributable to dietary risks by SDI regions in 2021. (A) ASMR. (B) ASDR. (C) Age-standardized YLDs rate. (D) Age-standardized YLLs rate.

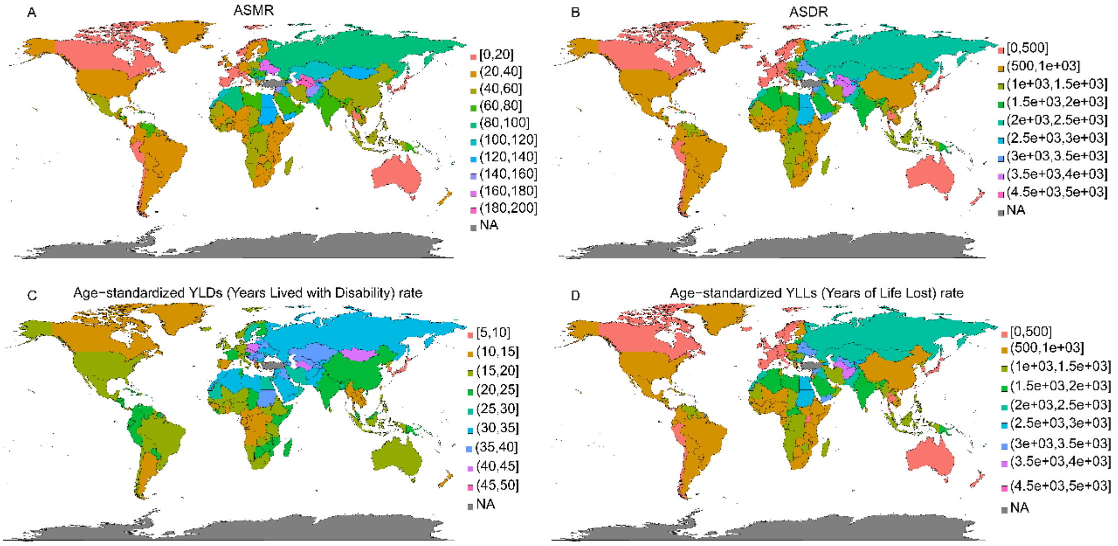


Figure S5 The burden of IHD attributable to dietary risks in 204 countries and territories in 2021. (A) ASMR. (B) ASDR. (C) Age-standardized YLDs rate. (D) Age-standardized YLLs rate.

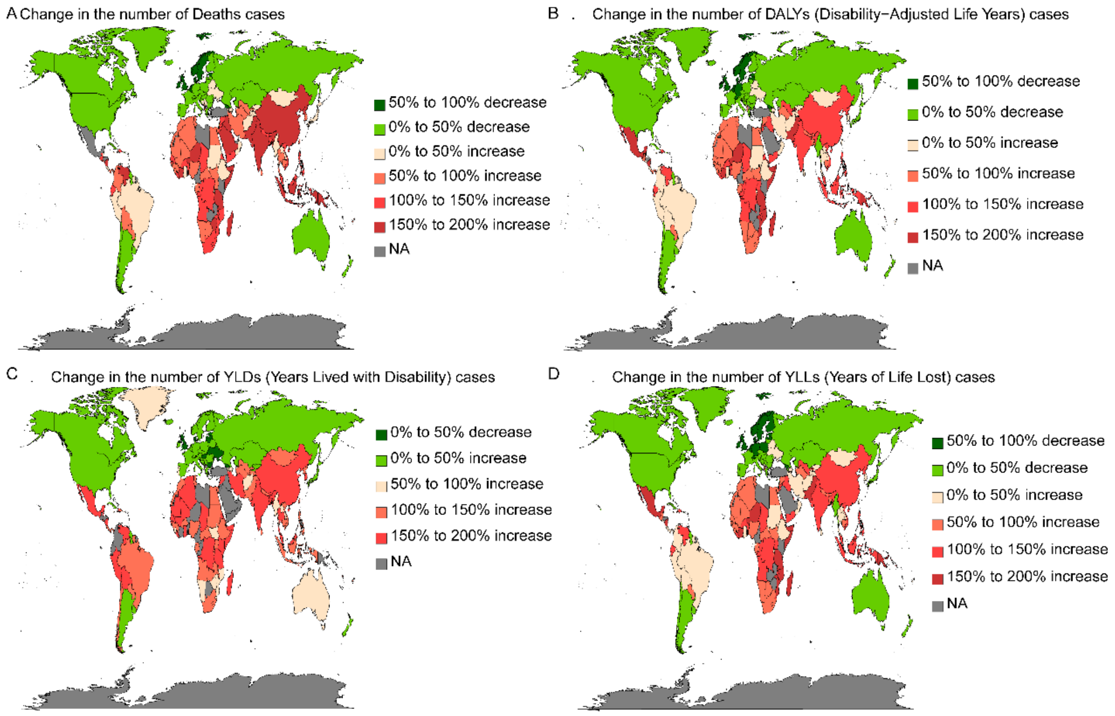


Figure S6 The burden of IHD attributable to dietary risks in 204 countries and territories in 2021. (A) Changes in the number of deaths. (B) Changes in the number of DALYs. (C) Changes in the number of YLDs. (D) Changes in the number of YLLs.

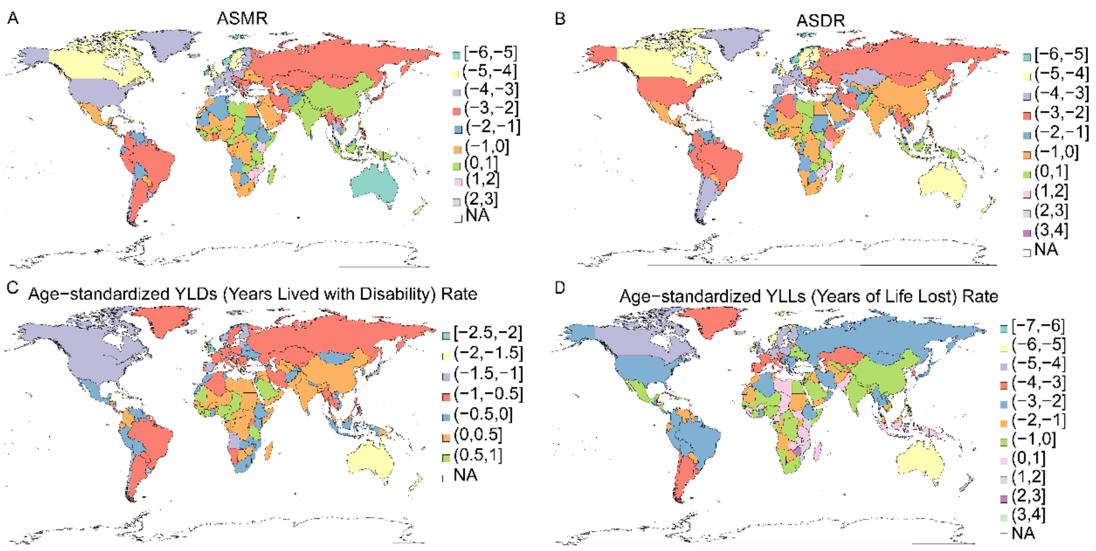


Figure S7 The burden of IHD attributable to dietary risks in 204 countries and territories in 2021. (A) EAPC of ASMR. (B) EAPC of ASDR. (C) EAPC of age-standardized YLDs rate. (D) EAPC of age-standardized YLLs rate.

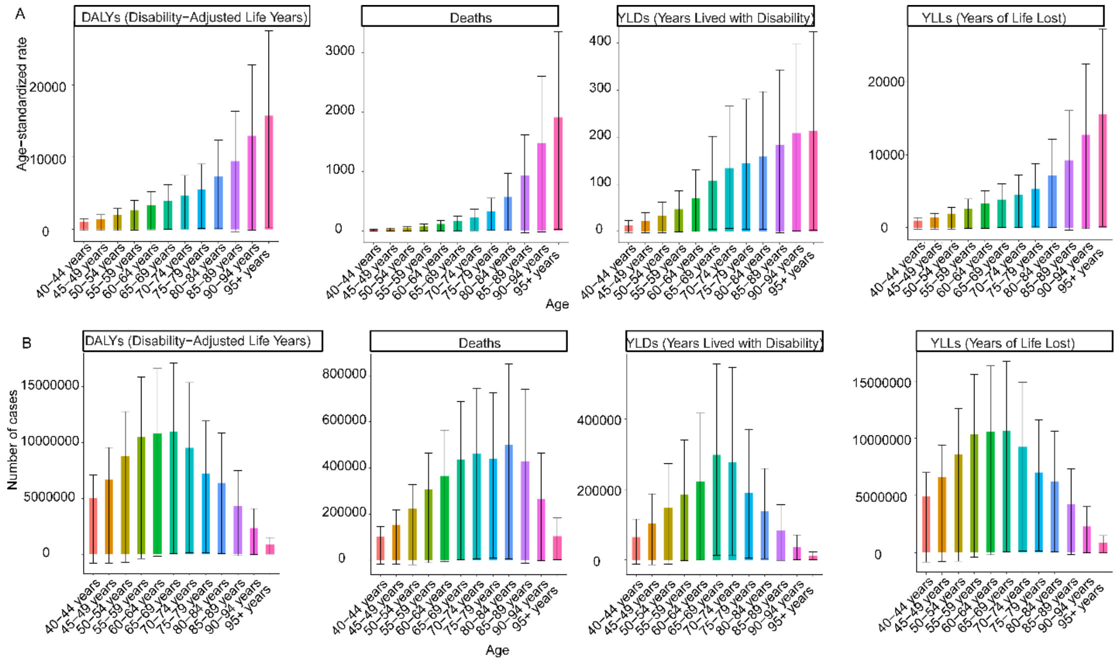


Figure S8 Age-specific impact of IHD attributable to dietary risks in 2021. (A) The number of deaths, DALYs, YLDs, and YLLs. (B) ASMR, ASDR, age-standardized YLDs rate, age-standardized YLLs rate.

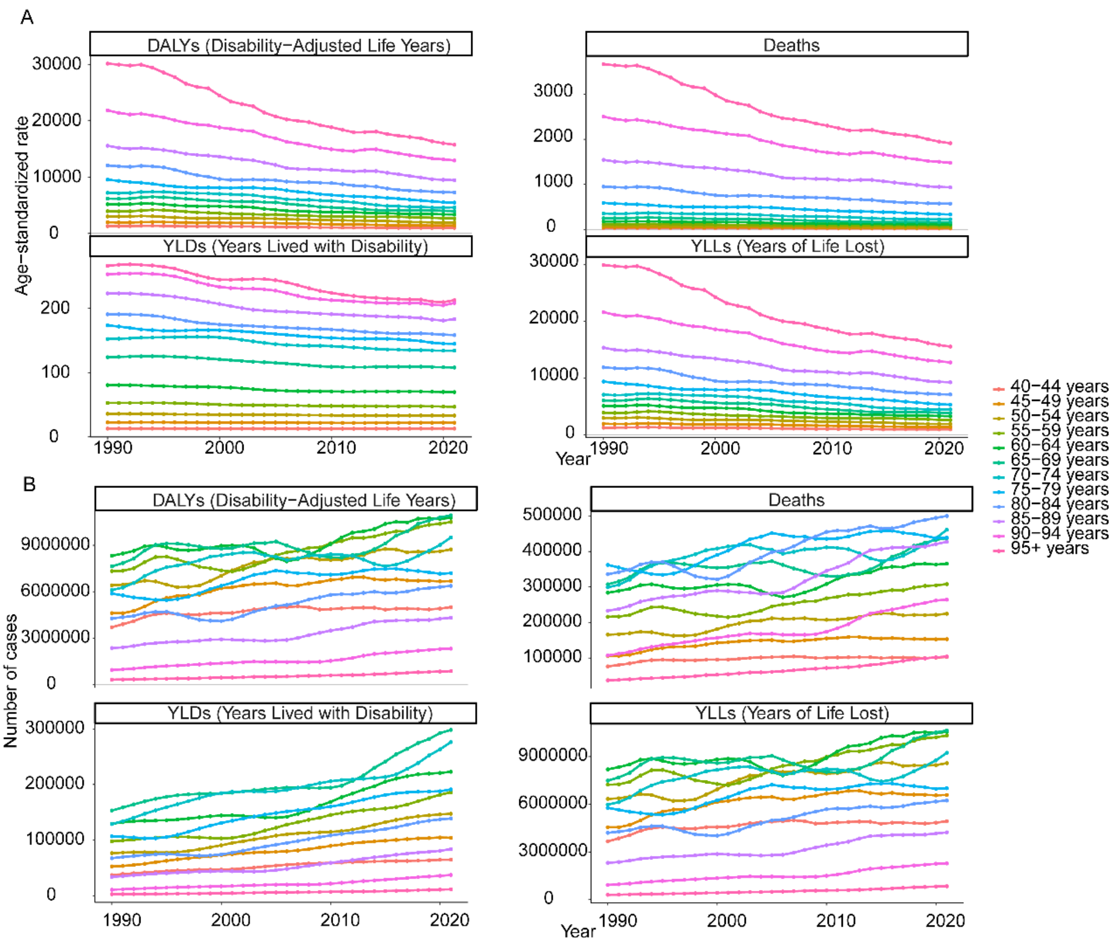


Figure S9 Trends in disease burden of IHD attributable to dietary risks by age group from 1990 to 2021. (A) The number of deaths, DALYs, YLDs, and YLLs. (B) ASMR, ASDR, age-standardized YLDs rate, age-standardized YLLs rate.

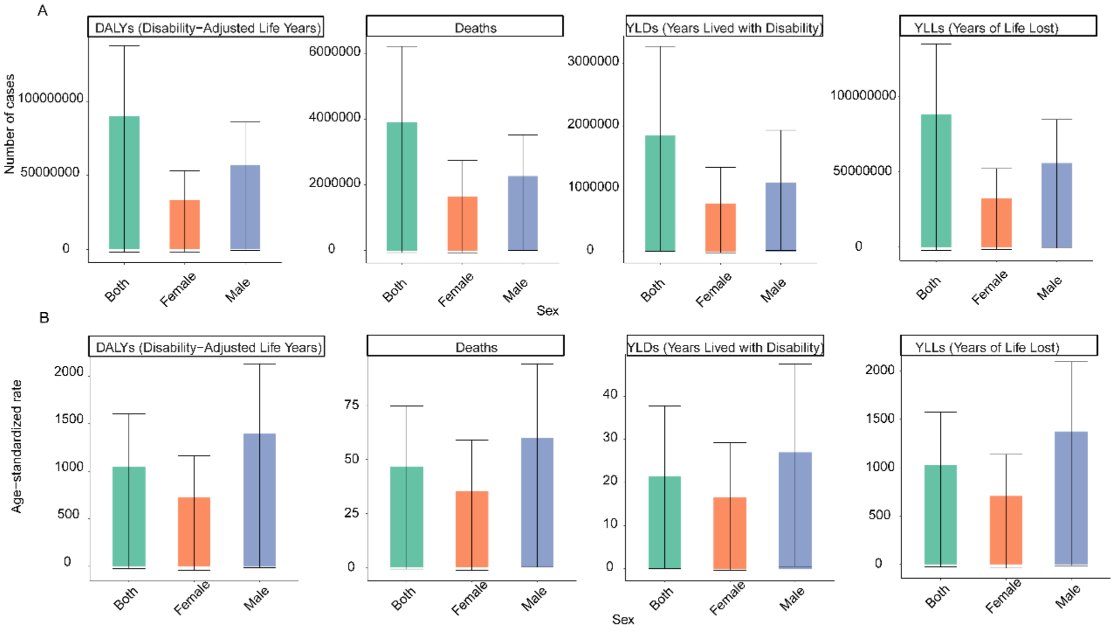


Figure S10 Gender disparities in the burden of IHD attributable to dietary risks in 2021. (A) The

number of deaths, DALYs, YLDs, and YLLs. (B) ASMR, ASDR, age-standardized YLDs rate, age-standardized YLLs rate.

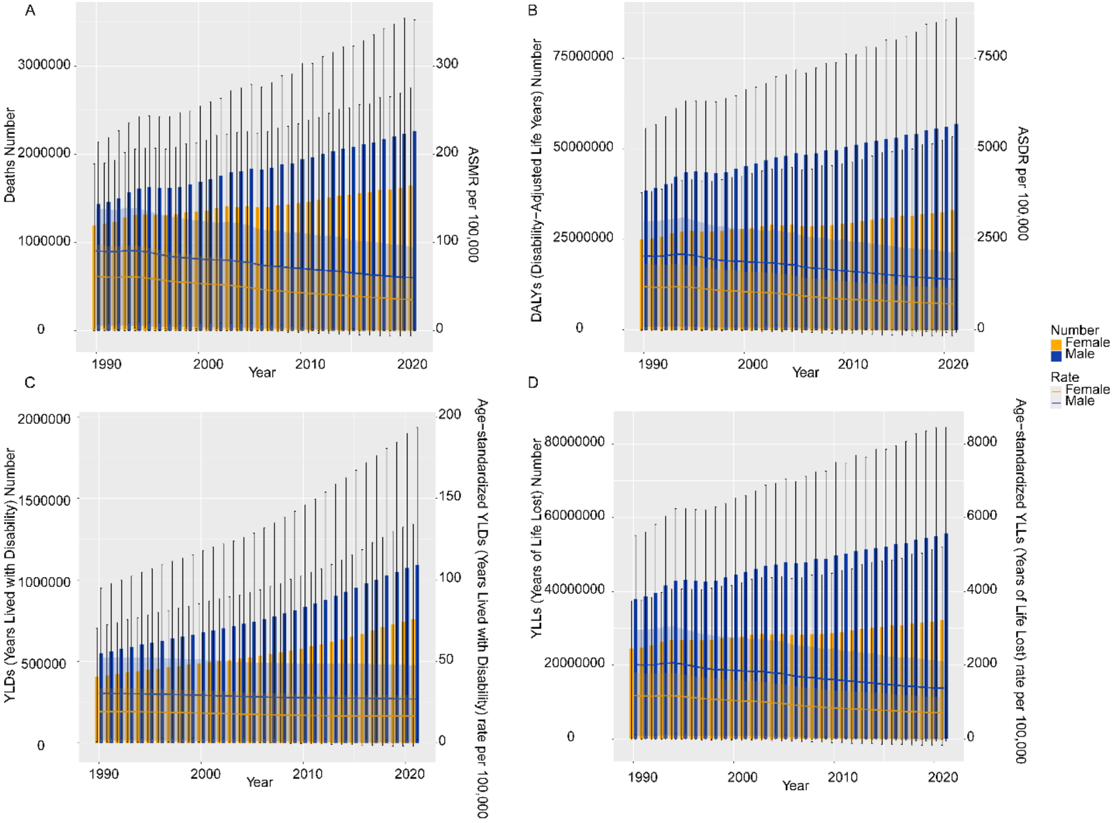


Figure S11 Trends of IHD attributable to dietary risks by sex from 19990 to 2021. (A) Deaths and ASMR. (B) DALYs cases and ASDR. (C) YLDs cases and age-standardized YLDs rate. (D) YLLs cases and age-standardized YLLs rate.

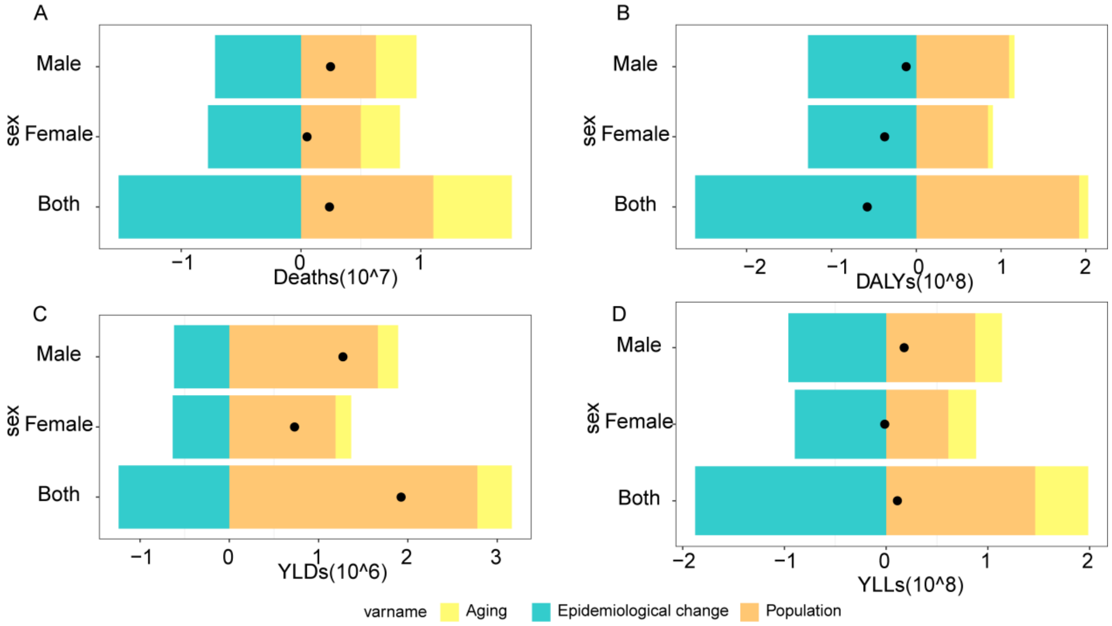


Figure S12 Changes in IHD attributable to dietary risks according to population-level determinants

of population growth, aging, and epidemiological change by sex. (A) Deaths. (B) DALYs cases. (C) YLDs cases. (D) YLLs cases.
